# High prevalence of colistin heteroresistance in *Pseudomonas aeruginosa* bloodstream isolates and associated clinical factors

**DOI:** 10.1007/s10096-026-05576-4

**Published:** 2026-06-24

**Authors:** Ekin Kırbaş, Muhammed Cihan Işık, Rashad Ismayilov, Serhat Ünal, Banu Sancak

**Affiliations:** 1 Department of Medical Microbiology, Faculty of Medicine, Eskisehir Osmangazi University, Prof. Dr. Nabi Avcı Boulevard, Meselik Campus, Eskisehir, 26040 Turkey; 2 Department of Clinical Microbiology and Infectious Diseases, Faculty of Medicine, Hacettepe University, Hacettepe Mah., Adnan Saygun Cad., Sıhhiye, Altındağ, Ankara, 06230 Turkey; 3 Department of Internal Medicine, Faculty of Medicine, Hacettepe University, Hacettepe Mah., Adnan Saygun Cad., Sıhhiye, Altındağ, Ankara, 06230 Turkey; 4 Department of Medical Microbiology, Faculty of Medicine, Hacettepe University, Hacettepe Mah., Adnan Saygun Cad., Sıhhiye, Altındağ, Ankara, 06230 Turkey

**Keywords:** *Pseudomonas aeruginosa*, Bloodstream infections, Colistin heteroresistance, Clinical impact

## Abstract

**Purpose:**

Carbapenem-resistant *Pseudomonas aeruginosa* (CRPA) has been classified by the World Health Organization as one of the most alarming antimicrobial-resistant microorganisms. Despite the availability of newer therapeutic options, polymyxins remain important last-resort agents for the treatment of CRPA infections in many settings. With the extended use of polymyxins in the treatment of extensively drug-resistant bacterial infections, both colistin resistance rates, colistin heteroresistance (CHR) rates and antibiotic treatment failures have risen. In this study, we aimed to: (i) determine the prevalence of CHR in *P. aeruginosa* bloodstream isolates, (ii) compare the accuracy of the antibiotic gradient test (GT) with the population analysis profiling (PAP) method, and (iii) identify clinical risk factors for CHR.

**Methods:**

CHR was investigated among 70 colistin-susceptible *P. aeruginosa* strains isolated from blood cultures using PAP and GT methods. Clinical and demographical data were retrospectively reviewed, and risk factors for CHR were analyzed using logistic regression.

**Results:**

We detected a remarkably high prevalence of CHR (61.4%) among colistin-susceptible *P. aeruginosa* bloodstream isolates. The gradient test failed to detect any of the heteroresistant strains. Our analysis revealed that age ≥ 60 years, lack of recent hospitalization, and a colistin MIC of 1 mg/L, compared with 2 mg/L, were independently associated with an increased risk of CHR. CHR was not significantly associated with 30-day or 90-day mortality.

**Conclusion:**

Despite the microbiological importance of heteroresistance, the clinical impacts and underlying mechanisms of this phenotype remain to be elucidated.

## Introduction

*Pseudomonas aeruginosa* stands out as a formidable pathogen, causing serious nosocomial infections associated with substantial morbidity and mortality. The World Health Organization (WHO) has classified carbapenem-resistant *P. aeruginosa* (CRPA) as a critical priority pathogen because of the limited therapeutic options available for its treatment [1]. Managing *P. aeruginosa* infections poses significant challenges due to its diverse intrinsic resistance mechanisms and remarkable capacity to acquire additional resistance determinants [2]. Importantly, bloodstream infections represent one of the most severe manifestations of *P. aeruginosa* infection and are associated with high mortality rates.

Although current guidelines generally favor newer β-lactam/β-lactamase inhibitor combinations over polymyxins for the treatment of CRPA infections [3], polymyxins remain important last-resort agents in many resource-limited settings where access to these newer therapies is restricted [4]. Under these conditions, with the increased rates of multi-drugresistant *P. aeruginosa* and CRPA infections, polymyxins often remain among the few available therapeutic options despite their well-recognized toxicity [5].

Heteroresistance refers to the presence of resistant subpopulations within an phenotypically susceptible bacterial isolate [6, 7]. Conventional susceptibility testing methods, including broth microdilution (BMD), may fail to detect heteroresistance because resistant subpopulations are often present at low frequencies within the susceptible main population [6, 7]. This phenomenon may facilitate bacterial survival during antimicrobial exposure and contribute to treatment failure despite susceptibility results indicating activity of the administered agent. Furthermore, resistant subpopulations may rapidly become dominant during therapy, potentially resulting in microbiological failure despite initial susceptibility results [8]. Despite the microbiological importance of heteroresistance, the clinical impacts and underlying mechanisms of this phenotype remain to be elucidated.

Although CHR has been described in *P. aeruginosa*, reported prevalence rates vary substantially across studies, likely due to differences in study populations and heteroresistance definitions. In this study, we aimed to: (i) determine the prevalence of CHR in *P. aeruginosa* bloodstream isolates, (ii) compare the performance of the antibiotic gradient test with PAP for detecting CHR, and (iii) identify clinical risk factors associated with CHR.

## Materials and methods

### Bacterial strains

This single-center retrospective study was conducted at the 1200-bed Hacettepe University, Adult and Oncology Hospital in Ankara, Türkiye. Seventy non-duplicate colistin-susceptible *P. aeruginosa* strains were recovered from blood cultures sent to the Central Microbiology Laboratory of HUH from January 2014 to July 2018. From 2014 to 2017, the isolates were identified to the species level by using VITEK^®^ MS (bioMérieux, Marcy-l’Étoile, France) and as of 2018 by MALDI Biotyper^®^ (Bruker Diagnostics, Bremen, Germany). Strains with colistin MIC values of 1 and 2 mg/L were included in the study. Only one bacterial strain per patient was included. Demographic and clinical data of the patients were obtained from the medical records and laboratory databases in the hospital server system.

### Antibiotic susceptibility testing

Colistin and meropenem minimum inhibitory concentrations (MICs) were determined using the broth microdilution method. Colistin sulfate (Product No. C4461, Sigma–Aldrich, Germany) and meropenem (Product No. PHR1772, Sigma–Aldrich, Germany) powders were dissolved in sterile water according to the manufacturers’ instructions. Two-fold serial dilutions were prepared to obtain concentration ranges of 0.06–128 mg/L for colistin and 0.03–64 mg/L for meropenem. MIC results were interpreted according to the European Committee on Antimicrobial Susceptibility Testing clinical breakpoints (Version 15.0, 2025; breakpoints for *Pseudomonas* spp.: susceptible: MIC ≤ 2 mg/L; resistant: MIC > 2 mg/L) [9]. CHR in *P. aeruginosa* strains with colistin MICs of 1 mg/L and 2 mg/L was assessed using the PAP method.

### Population analysis profiling (PAP) method

For this purpose, Mueller-Hinton agar (MHA, Product No.: CM0337, Oxoid, United Kingdom) plates with 1, 2, 4, 8, 16, 32 and 64 mg/L colistin sulphate (Product No. PHR1772, Sigma–Aldrich) concentrations were prepared. For each isolate, serial 10-fold saline dilutions, ranging from 10^8^ to 10^2^ CFU/mL, were prepared from overnight cultures. 100 µL of each dilution was inoculated on MHA plates with two-fold serial concentrations of colistin. Plates were incubated at 35 ± 2 °C for 48 h, and colonies were counted at 24 and 48 h. HR was considered, if the ratio of the lowest colistin concentration exhibiting maximum inhibition to the highest non-inhibitory colistin concentration was equal or more than eight-fold, with the frequency of resistant subpopulation in a bacterial colony at least 1 × 10^− 7^ CFU/mL [5, 6]. The frequency of resistant subpopulations was determined by dividing the number of *P. aeruginosa* colonies grown on the MHA plate with the highest colistin concentration to the number of the colonies on the antibiotic-free plate. *Escherichia coli* ATCC 25,922 and *E. coli* NCTC 13,846 were included as quality-control strains during PAP experiments.

### Antibiotic gradient test method

Bacterial suspensions with a 0.5 McFarland standard were spread on MHA plates using a sterile cotton swab. Colistin gradient test strips (bioMérieux, Marcy-l’Étoile, France) were placed on the inoculated plates. After incubation at 35 ± 2 °C for 48 h, the presence of bacterial growth within the inhibition zone of the colistin GT strip was examined.

### Defining the stability of the heteroresistance

To evaluate the stability of the HR phenotype colonies from PAP plates with the highest drug concentration were subcultured daily in an antibiotic-free medium for seven consecutive days, following approaches commonly used in previous heteroresistance studies. Colistin BMD was performed on days 1 and 7 to assess CHR stability. Isolates remaining resistant after 7 days were classified as ‘*stable CHR*,’ while those becoming susceptible were ‘*unstable CHR*.’

### Statistical analysis

The statistical analysis of our study was performed using the Statistical Package for the Social Sciences software (IBM SPSS Statistics, Version 26). The median (interquartile range [IQR]) was used for non-normally distributed data. Categorical variables were expressed as percentages. Continuous variables were compared using the Mann–Whitney U test. Fisher’s exact test and the chi-squared test were used for categorical comparisons. To identify risk factors associated with CHR, a univariate logistic regression analysis was first performed. Variables with a *p*-value < 0.25 in the univariate analysis or considered clinically relevant were included in the multivariable model. Multicollinearity among independent variables was assessed using the Phi coefficient, with a correlation coefficient (r) > 0.60 considered indicative of significant multicollinearity. Additionally, the Variance Inflation Factor (VIF) was utilized to evaluate multicollinearity in the final multivariable model and all variables in the final model had a VIF value of less than 2, indicating no significant multicollinearity. Potential interactions between predictors were examined using the Breslow-Day test, while confounding effects were evaluated with the Mantel-Haenszel test. The final multivariable risk model was constructed using the backward stepwise likelihood ratio method. The overall significance of the model was assessed with the Omnibus Test of Model Coefficients, and a *p-*value < 0.05 indicated that the model was statistically significant. Model fit was evaluated using the Hosmer–Lemeshow test, with a *p*-value > 0.05 suggesting good fit to the data. Nagelkerke R² was used to assess the explanatory power of the model, and − 2 log likelihood (–2LL) was reported as a measure of model fit. Results were presented as odds ratios (ORs) with 95% confidence intervals (CIs), with multivariable results expressed as Adjusted Odds Ratios (aORs). Survival analysis for 30-day and 90-day mortality was performed using the Kaplan-Meier method, and survival distributions between the groups were compared using the log-rank test. A *p*-value of less than 0.05 was considered as statistically significant.

## Results

### Population analysis profiling results

**Fig. 1:**
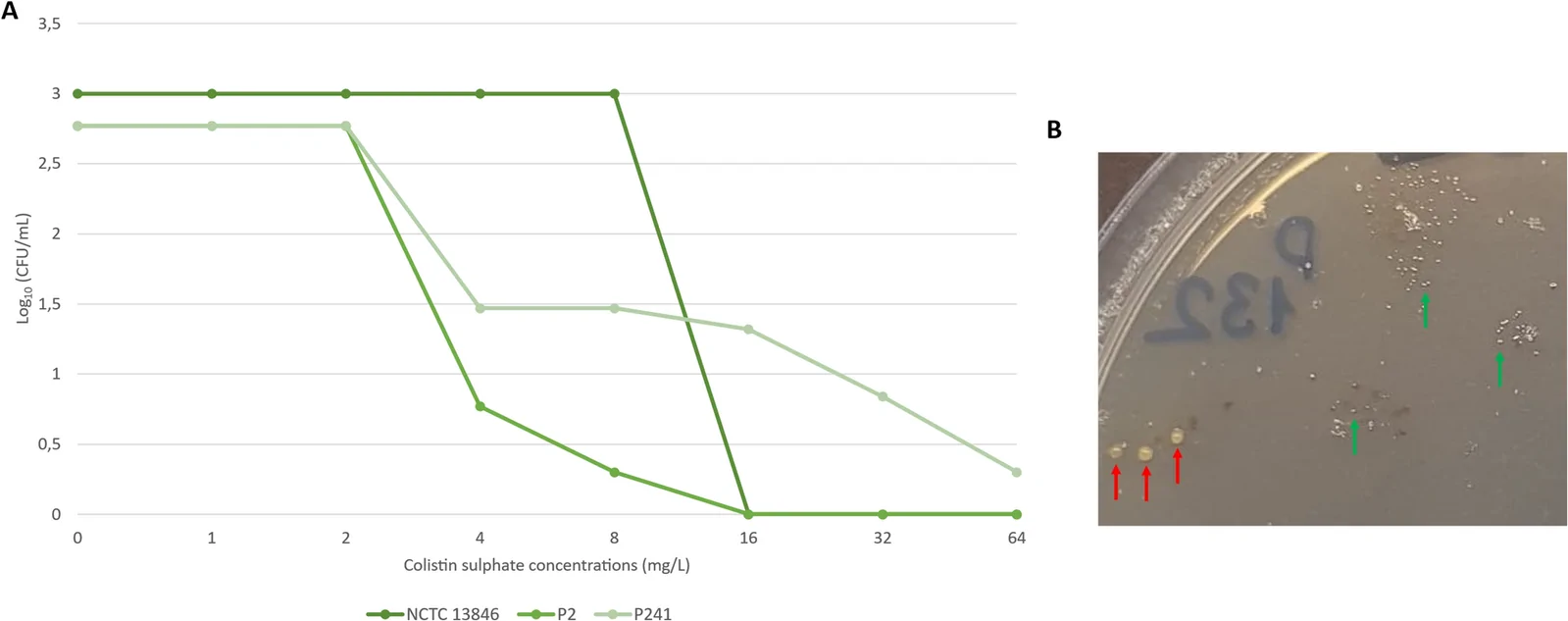
**(A)** PAP curves of two selected colistin heteroresistant strains (P2, P241) and *E. coli* NCTC 13846, **(B)** Colistin heteroresistant *P. aeruginosa* isolates showing large and small colonies on PAP plate (red arrow: large colonies, green arrows: small colonies). (CFU/mL, Colony forming units per milliliter; log_10_, logarithm with a base equal to 10; mg/L, milligrams per liter

CHR was detected in 43 of 70 (61.4%) of the colistin-susceptible *P. aeruginosa* strains. The prevalence of CHR was 68.6% (*n* = 35) among the isolates with a colistin MIC of 1 mg/L and 42.1% (*n* = 8) among the isolates with a colistin MIC of 2 mg/L. The frequency of the colistin-resistant subpopulations in the main colistin-susceptible populations ranged from 1.1 × 10^− 7^ to 6.0 × 10^− 6^ CFU/mL. The PAP curves of CHR *P. aeruginosa* strains demonstrated a slight shift to the right at higher colistin concentrations (Fig. 1a). In one of the strains (Isolate-P132), we detected subpopulations with different morphologies (small and large colonies) on PAP plates containing high colistin concentration (Fig. 1b). No growth occurred within the inhibition zone of the colistin GT strips for any of the isolates. Table 1 shows the PAP results. ‘Skipped wells’ phenomenon, which is described as the absence of bacterial growth at lower antibiotic concentrations of BMD microplate, but the presence of bacterial growth at higher antibiotic concentrations, and linked to HR phenotype, was not detected with any of the isolates.

**Table 1:** The colistin MIC values, and PAP results of colistin-heteroresistant *P. aeruginosa* strains

Isolate No.	Colistin MIC of the main pop.	The max. antibiotic con. of the PAP plate growth observed	Growth within the inhibiton zone of GT	Frequency of the subpop. (CFU/mL)	Colistin MICs after 7 days of cultivation
Colistin MIC: 1 mg/L					
P2	1	8	-	4.2 × 10^− 7^	2
P18	1	8	-	1.3 × 10^− 7^	4
P21	1	16	-	2.1 × 10^− 7^	2
P36	1	8	-	2.5 × 10^− 7^	4
P46	1	4	-	1.5 × 10^− 7^	2
P59	1	32	-	6.5 × 10^− 7^	16
P68	1	8	-	2.5 × 10^− 7^	2
P96	1	8	-	6.7 × 10^− 7^	2
P139	1	32	-	3.7 × 10^− 7^	4
P141	1	32	-	1.3 × 10^− 7^	1
P143	1	16	-	7.5 × 10^− 7^	2
P149	1	32	-	1 × 10^− 6^	2
P154	1	16	-	8.7 × 10^− 7^	4
P157	1	8	-	4 × 10^− 7^	8
P162	1	16	-	1.2 × 10^− 6^	4
P165	1	16	-	5.3 × 10^− 7^	4
P166	1	4	-	2 × 10^− 7^	2
P167	1	8	-	6.3 × 10^− 7^	2
P172	1	8	-	1.7 × 10^− 7^	8
P182	1	64	-	1.7 × 10^− 7^	2
P187	1	8	-	1.5 × 10^− 7^	1
P188	1	64	-	1.2 × 10^− 7^	128
P189	1	8	-	4.4 × 10^− 7^	8
P190	1	8	-	1.5 × 10^− 7^	1
P191	1	4	-	3.8 × 10^− 7^	0.5
P193	1	16	-	2 × 10^− 7^	16
P194	1	64	-	1.5 × 10^− 6^	16
P210	1	16	-	6 × 10^− 6^	1
P212	1	4	-	1.1 × 10^− 7^	1
P216	1	8	-	1.8 × 10^− 7^	0.5
P219	1	8	-	1.3 × 10^− 7^	1
P221	1	16	-	1.3 × 10^− 7^	1
P235	1	16	-	2.0 × 10^− 7^	16
P236	1	16	-	1.1 × 10^− 7^	4
P241	1	64	-	1.3 × 10^− 7^	2
Colistin MIC: 2 mg/L					
P115	2	4	-	1 × 10^− 7^	1
P119	2	8	-	1.5 × 10^− 7^	4
P132	2	32	-	6.6 × 10^− 7^	16*
					0.5**
P146	2	8	-	3 × 10^− 7^	1
P180	2	8	-	1.5 × 10^− 7^	2
P185	2	32	-	1.1 × 10^− 7^	8
P197	2	8	-	1.5 × 10^− 7^	2
P237	2	16	-	2.5 × 10^− 7^	4

CFU/mL, colony-forming unit per milliliter; con., concentration; GT, gradient test; mg/L, milligrams per liter; MIC, minimum inhibitory concentration; *No.*, numero; pop., population; *, small colony morphology; subpop., subpopulation; **; large colony morphology; -, absent

### Stability of heteroresistance

After seven consecutive subculturing of the colistin-resistant subpopulations on 5% sheep blood agar plates, the colistin BMD test showed stable CHR in 54.2% of isolates and unstable CHR in 45.8%. Following a week-long cultivation of small and large colonies of Isolate-P132 on an antibiotic-free medium, it was observed that colonies exhibiting diverse morphologies possessed varying minimum inhibitory concentrations. Small colonies had a colistin MIC of 16 mg/L (colistin-resistant), while large colonies had a MIC of 0.5 mg/L (COL-susceptible).

### Demographic and clinical features of CHR-positive and CHR-negative patients

Among patients, 31 (44%) were female and the median age was 60 years (IQR: 50–71). Before the detection of *P. aeruginosa* bacteremia, the CHR-positive group had a significantly higher median age (65 years; IQR 55–74) than the CHR-negative group (*p* = 0.003). The median Charlson comorbidity index was three with no significant difference between the two groups (Table 2). No significant difference was observed between the CHR-positive and CHR-negative groups in the presence of severe immunosuppressive status, history of invasive interventional procedures, and history of ICU admission. However, hospitalization within the past three months was lower in CHR-positive cases (*p* = 0.05).

**Table 2:** Clinical characteristics of patients with colistin susceptible *P. aeruginosa* in blood isolates grouped by colistin heteroresistance status

Characteristics *n* (%)	Total, *n* = 70	CHR-Negative, *n* = 27	CHR-Positive, *n* = 43	*P* value
Female sex	31 (44.3)	11 (40.7)	20 (46.5)	0.636
Age, median (IQR)	60 (50–71)	54 (43–61)	65 (55–74)	**0.003**^**a**^
Grouped Age				**0.004**^**b**^
< 60 ≥ 60	34 (48.6) 36 (51.4)	19 (70.4) 8 (29.6)	15 (34.9) 28 (65.1)	
CCI, median (IQR)	3 (0–10)	4 (0–10)	3 (0–9)	0.478
Severe Immunocompromised status	19 (27.1)	6 (22.2)	13 (30.2)	0.463
Prolonged Neutropenia	10 (52.6)	1 (16.7)	9 (69.2)	
HSCT	6 (31.6)	4 (66.7)	2 (15.4)	
SOT	3 (15.8)	1 (16.7)	2 (15.4)	
Invasive intervention history (past 3 months)	39 (55.7)	15 (55.6)	24 (55.8)	0.983
Hospitalisation history (past 3 months)	39 (55.7)	19 (70.4)	20 (46.5)	**0.050**
ICU Admission (past 3 months)	14 (20.0)	7 (25.9)	7 (16.3)	0.326
Pseudomonal colonisation (past 6 months) Non-MDR MDR XDR	9 (14.8) 7 (77.8) 1 (11.1) 1 (11.1)	4 (18.2) 3 (75) 0 (0) 1 (25)	5 (12.8) 4 (80) 1 (20) 0 (0)	0.710
Antibiotic history (past 3 months)	45 (72.6)	19 (82.6)	26 (66.7)	0.174
Anti-pseudomonal antibiotic history (past 3 months)	38 (61.3)	16 (69.6)	22 (56.4)	0.304
Colistin history (past 3 months)	13 (21)	5 (21.7)	8 (20.5)	1.000
Source of infection				0.895
Pneumonia Primer bacteraemia Intrabdominal Urinary Soft tissue Other	23 (32.9) 18 (25.7) 13 (18.6) 6 (8.6) 5 (7.1) 5 (7.1)	8 (29.6) 8 (29.6) 4 (14.8) 2 (7.4) 3 (11.1) 2 (7.4)	15 (34.9) 10 (23.3) 9 (20.9) 4 (9.3) 2 (4.7) 3 (7)	
Presence of sepsis	37 (52.9)	16 (59.3)	21 (48.8)	0.395
Meropenem Resistance	30 (42.9)	13 (48.1)	17 (39.5)	0.478
Colistin MIC (mg/L)				**0.043**^**b**^
1 2	51 (72.9) 19 (27.1)	16 (59.3) 11 (40.7)	35 (81.4) 8 (18.6)	
Anti-pseudomonal antibiotics administered	64 (91.4)	25 (92.6)	39 (90.7)	0.783
In-vitro active antibiotics administered	55 (78.6)	21 (77.8)	34 (79.1)	0.898
Colistin-based therapy	13 (18.6)	4 (14.8)	9 (20.9)	0.522
Colistin monotherapy	3 (23.1)	1 (25)	2 (22.2)	0.913
Colistin duration, median (IQR)	8 (2–13)	7.5 (4–9.5)	12 (1–15.5)	0.938
Clinical response (*n* = 62)*	32 (51.6)	8 (34.8)	24 (61.5)	**0.042**^**b**^
Microbiological outcome				0.153
Persistent Eradicated Undetermined	3 (4.3) 27 (38.6) 40 (57.1)	2 (7.4) 7 (25.9) 18 (66.7)	1 (2.3) 20 (46.5) 22 (51.2)	
Mortality, 30-day	39 (55.7)	18 (66.7)	21 (48.8)	0.144
Mortality, 90-day	44 (62.9)	21 (77.8)	23 (53.5)	**0.041**^**b**^

CHR, colistin heteroresistance; IQR, interquartile range; CCI, Charlson comorbidity index; HSCT, hematopoietic stem cell transplantation; SOT, solid organ transplantation; ICU, intensive care unit; MDR, multiple drug-resistance; XDR, extensively drug-resistant; MIC, minimal inhibitory concentration

^a^Mann-Whitney *U*-test

^b^χ^2^ test or Fisher’s exact test

*Clinical response was assessed in a total of 62 patients who received anti-pseudomonal treatment and who could be followed up (two patients were transferred to another hospital)

Nine patients (14.8%) had a history of *P. aeruginosa* colonization within six months. Additionally, 38 patients (61.3%) had received anti-pseudomonal antibiotics within the last three months, and 13 patients (21%) had a history of colistin use. There was no significant difference between the two groups regarding prior exposure to anti-pseudomonal antibiotics or colistin.

At the time of *P. aeruginosa* bacteremia detection, 52.9% of the patients were in sepsis. Pneumonia (*n* = 23, 32.9%) and primary bacteremia (*n* = 18, 25.7%) were found as the source of bacteremia in more than half of the patients. Anti-pseudomonal treatment was initiated in 64 patients (91.4%). Two of these patients were transferred to another facility; therefore, only mortality data were available for them. Overall, 78.6% (*n* = 55) of the patients received in vitro active treatment, with comparable rates between the CHR-positive and CHR-negative groups. Colistin-based regimens were initiated in 13 patients, with a similar distribution between the two groups. The overall clinical response rate was 51.6% (*n* = 32), which was significantly higher in the CHR-positive group (*p* = 0.042). While 30-day mortality rates were comparable between the groups, chi-square analysis revealed a lower 90-day mortality rate in the CHR-positive group (*p* = 0.041). However, log-rank analysis demonstrated no significant difference in 30 and 90-day survival distributions between patients with and without CHR, respectively (*p* = 0.693 and *P* = 0.448, respectively). Microbiological response could not be evaluated in 40 (57.1%) patients due to a lack of follow-up cultures; among the remaining patients, microbiological eradication was confirmed in 27.

Among the 30 patients with meropenem-resistant isolates, 17 (56.7%) were CHR-positive, and 10 (33.3%) had received a colistin-containing regimen. In this subgroup, the clinical response rate was 57.1% in CHR-positive cases compared to 33.3% in CHR-negative cases (*p* = 0.225). The 30-day and 90-day mortality rates were both 58.8% in the CHR-positive group, compared to 69.2% and 76.9% in the CHR-negative group, respectively. However, no statistically significant difference was observed in the 30-day and 90-day mortality rates between these two groups (*p* = 0.708 and *p* = 0.440, respectively).

In the CHR-positive group, 81.4% of isolates had a colistin MIC of 1 mg/L, whereas this rate was 59.3% in the CHR-negative group. This difference was statistically significant (*p* = 0.043). Carbapenem resistance rates were similar between CHR-positive (39.5%) and CHR-negative (48.1%) groups (*p* = 0.478).

### Risk factors for CHR

Table 3 summarizes the results of univariable and multivariable logistic regression analyses assessing clinical factors associated with CHR. In the final multivariable model, age ≥ 60 years (aOR: 9.222, 95% CI: 2.229–38.154, *p* = 0.002), lack of hospitalization in the past three months (aOR: 6.285, 95% CI: 1.480–26.690, *p* = 0.013), and a colistin MIC of 1 mg/L compared to 2 mg/L (aOR: 5.490, 95% CI: 1.165–25.877, *p* = 0.031) were independently associated with CHR. Colistin history in the past three months was also included in the multivariable model due to its confounding effect, although it did not show a statistically significant independent association with CHR (aOR: 1.929, 95% CI: 0.389–9.578, *p* = 0.422). Other evaluated variables, including prior antibiotic use, and anti-pseudomonal antibiotic history were not significantly associated with CHR.

**Table 3:** Clinical risk factors for CHR in a logistic regression model

Variable	Univariable		Multivariable	
	OR (95% CI)	*P* value	aOR (95% CI)	*P* value
Age (years)	1.036 (1.006–1.067)	**0.020**	-	**-**
Age ≥ 60	4.433 (1.571–12.507)	**0.005**	9.222 (2.229–38.154)	**0.002**
No hospitalization history (past 3 months)	2.731 (0.984–7.578)	0.054	6.285 (1.480–26.690)	**0.013**
Antibiotic history (past 3 months)	0.421 (0.119–1.495)	0.181	-	-
Anti-pseudomonal antibiotic history (past 3 months)	0.566 (0.190–1.685)	0.307	-	-
Colistin history (past 3 months)	0.929 (0.264–3.273)	0.909	1.929 (0.389–9.578)	0.422
Colistin MIC (1, compared to 2 mg/L)	3.008 (1.015–8.910)	**0.047**	5.490 (1.165–25.877)	**0.031**

CI, confidence interval; MIC, minimum inhibitory concentration (mg/L); OR, odds ratio; aOR, adjusted OR; bold texts indicate the statistically significant *p*-values

Omnibus Test of Model Coefficients chi-square = 23.390, df = 4, *P* < 0.001; Hosmer–Lemeshow test *P* = 0.970; Nagelkerke R² = 0.429; all VIF values ranged between 1.010 and 1.022

## Discussion

Within this conceptual framework, our findings may also be interpreted in light of the emerging paradigm of antimicrobial heteroresistance. In our study, we detected a remarkably high prevalence of CHR (61.4%) among COL-susceptible *P. aeruginosa* bloodstream isolates using the PAP method, considerably higher than the results of previous studies conducted. Notably, the GT failed to identify any heteroresistant strains, highlighting the limitations of conventional susceptibility testing methods in detecting minority resistant subpopulations. Furthermore, since there are limited studies investigating the effect of HR on clinical outcomes, in the present study, we found that age ≥ 60, lack of hospitalization history, and a colistin MIC of 1 mg/L were risk factors for CHR. However, no significant association was observed between CHR and mortality.

The remarkably high prevalence of CHR observed in our study warrants careful interpretation. Several methodological and epidemiological factors may have contributed to this finding, including differences in heteroresistance definitions and PAP interpretation criteria. Previous studies have applied different resistant-subpopulation frequency thresholds (e.g., 10⁻⁶ versus 10⁻⁷), different concentration cut-offs, and different PAP interpretation approaches. Consequently, direct comparison of CHR prevalence across studies should be made with caution, and the prevalence observed in our study may not be directly comparable to previously published reports. Although the prevalence observed in our study was higher than most previously reported rates, relatively high CHR frequencies have also been reported in studies using PAP-based methodologies [10]. This observation may suggest that at least part of the variability across studies reflects true epidemiological differences in addition to methodological heterogeneity. Nevertheless, the lack of an internationally standardized definition of colistin heteroresistance remains a major challenge for cross-study comparisons and highlights the need for consensus criteria for CHR detection. The available literature on colistin heteroresistance in *P. aeruginosa* is summarized in Table 4.

**Table 4:** Summaries of studies on colistin heteroresistance in *P. aeruginosa* isolates

Country	Clinical sample	Number of samples	Method for determination of HR	Prev. of CHR (%)	Ref.
Brasil	VCS	24	PAP (colonies growing on polymyxin B > 2 mg/L) and with an increase of 8 x MIC of the main pop.	4.2	[11]
			PAP (colonies growing on polymyxin B *≤* 2 mg/L) and with >2xMIC increase compared with the main pop.	37.5	
Hungary	Blood	76	Agar screen (colonies growing on colistin concent. of 4 mg/L)	27	[12]
China	VCS	231	PAP (colonies growing on colistin *≥* 2 mg/L)	3.9	[13]
Sweden	VCS	143	PAP (colonies growing on 4 and 8 mg/L of colistin), at a frequency of *≥*1 × 10^− 6^	6	[14]
			PAP (colonies growing on colistin concent. *≥*8xMIC of the main pop.), at a frequency of *≥*1 × 10^− 7^	26	
Iran	VCS	100	PAP (colonies growing on colistin concent. *≥*8xMIC of the main pop.), at a frequency of *≥*1 × 10^− 7^	8	[15]
Turkey	Blood	70	PAP (colonies growing on colistin concent. *≥*8xMIC of the main pop.), at a frequency of *≥*1 × 10^− 7^	61.4	The present study
			GT (bacterial growth within the inhibition zone)	0	

In addition to methodological variability, epidemiological characteristics of the study population and local antimicrobial selection pressures may also have influenced the observed CHR prevalence. Only 21% of patients had documented prior colistin exposure, suggesting that previous colistin therapy alone cannot explain the high prevalence of CHR observed in our study. The high prevalence of CHR may be influenced by factors beyond individual antibiotic exposure. Clinically achievable plasma colistin concentrations are limited by toxicity concerns and may not completely suppress resistant-subpopulations, potentially facilitating their persistence [5]. Furthermore, sustained colistin use in our hospital setting may have contributed to the maintenance and circulation of heteroresistant strains within the healthcare environment. These factors may partly explain the high frequency of CHR detected among bloodstream isolates in our study. Additionally, CHR has been reported more frequently among multidrug-resistant and carbapenem-resistant strains. Since our collection largely consisted of isolates recovered from a tertiary-care hospital with a high burden of resistant Gram-negative bacteria, the high CHR rate observed in our study may be associated with the predominance of resistant hospital lineages rather than prior colistin exposure alone [16]. The markedly lower CHR prevalence reported in the recent Iranian study may further illustrate the substantial variability observed across studies [15]. In addition to differences in PAP interpretation criteria, the two studies differed considerably in isolate selection. While the Iranian study included clinical isolates from various infection sites, our study was restricted to bloodstream isolates and predominantly included strains with colistin MICs of 1–2 mg/L, a factor independently associated with CHR in our cohort. Therefore, differences in study populations and isolate characteristics may have contributed to the discrepant prevalence estimates.

Another possible explanation for the high prevalence observed in our study is the composition of the isolate collection. Only bloodstream isolates with colistin MICs of 1–2 mg/L were included, and isolates with a MIC of 1 mg/L constituted the majority of the study population. Notably, a colistin MIC of 1 mg/L was independently associated with CHR, suggesting that the MIC distribution within the study sample may have contributed to the observed prevalance estimate. Since isolates with a MIC of 1 mg/L were independently associated with CHR in our cohort, the distribution of MIC values within the study population may have contributed to the observed prevalence estimate. This finding is noteworthy because it challenges the conventional assumption that higher MIC values necessarily indicate a greater likelihood of resistance-related phenomena. Instead, it suggests that isolates categorized as fully susceptible may still contain resistant subpopulations detectable by PAP. While the proportion of isolates with a colistin MIC of 1 mg/L in the study by Howard-Anderson et al. (66%, *n* = 95) was similar to that observed in our cohort, those authors did not identify a significant association between MIC category and CHR, in contrast to our findings [14].

The biological basis of stable and unstable heteroresistance remains incompletely understood. Stable heteroresistance may suggest the presence of heritable alterations that allow resistant subpopulations to maintain their phenotype after serial passages in antibiotic-free media. In contrast, unstable heteroresistance may reflect reversible adaptive responses to antibiotic exposure, including transient phenotypic switching or persistence-like states [6, 7]. Such mechanisms could permit survival during antibiotic treatment while not being permanently maintained in the bacterial population. Because the present study did not investigate the molecular mechanisms underlying heteroresistance stability, these interpretations should be considered speculative and require confirmation by future genomic and transcriptomic studies.

In the current study, the clinical response rate was statistically higher in the CHR-positive group than in the CHR-negative group (*p* = 0.042). It is crucial to highlight that the number of patients receiving colistin-based regimens in our cohort was highly limited (*n* = 13); therefore, this difference in clinical outcomes cannot be attributed to the choices or efficacy of colistin therapy. Importantly, this counterintuitive finding was not driven by baseline clinical disparities between the two groups. Major clinical determinants and confounding factors that directly influence prognosis—including the CCI, severe immunocompromised status, presence of sepsis at onset, meropenem resistance rates, the administration of in vitro active antibiotics, and colistin-based therapy—were highly comparable and showed no statistically significant differences between the CHR-positive and CHR-negative groups (Table 2). Given that these key clinical variables were well-balanced between the groups, the biological basis of the higher clinical response rate observed among CHR-positive patients remains unclear. One possible explanation may be the fitness cost associated with heteroresistance. However, this hypothesis was not directly evaluated in the present study and requires confirmation in future experimental investigations. Additionally, consistent with the findings of a previous study [14], no significant association was observed between CHR and overall mortality in our cohort. As with the clinical response rates, this lack of association cannot be attributed directly to the definitive clinical effect, or lack thereof, of the CHR phenotype, due to the small size of the subpopulation treated with colistin. Therefore, future prospective studies with larger cohorts of colistin-treated patients, complemented by molecular and genomic analyses, are warranted to fully elucidate the definitive clinical trajectory and underlying genetic mechanisms of colistin heteroresistance in bloodstream infections.

Another notable finding was the association between the absence of recent hospitalization and the presence of CHR. While hospitalization is traditionally considered a risk factor for antimicrobial resistance, this observation may indicate that heteroresistance is not solely driven by nosocomial selective pressures. This observation should be interpreted cautiously and validated in future studies. Furthermore, increasing age, which emerged as another independent predictor of CHR in our multivariable model, is a well-known risk factor for resistant *P. aeruginosa* infections [17].

Even though the PAP method is regarded as the most reliable method for detecting HR, its high cost and labor-intensive nature make it difficult implement in clinical settings. Therefore, diffusion-based tests are widely utilized in clinical microbiology laboratories, particularly in countries with a high prevalence of HR. In the present study, no bacterial growth was observed within the inhibition zone of colistin GT strips for any of the isolates identified as heteroresistant by the PAP method. Standard susceptibility testing methods may overlook clinically significant minority variants, potentially leading to underestimation of resistance and suboptimal treatment decisions. This limitation underscores the need for diagnostic approaches capable of capturing population-level complexity. This suggests that GT testing is insufficient for detecting CHR, a finding consistent with previous studies [18, 19]. The limited diffusibility of colistin on agar surfaces may account for the failure to detect CHR, similar with the limitations of diffusion-based techniques in determining colistin susceptibility.

The observation of distinct colony morphologies within heteroresistant subpopulations in one isolate further supports the concept of phenotypic diversification within bacterial populations. Although heteroresistance and small colony variant phenotypes are considered as distinct, our findings suggest that these two phenotypes may not be entirely separate concepts and could potentially overlap [20]. To our knowledge, no study in the literature presents data similar with our observation.

Our study has several limitations. First, it was conducted retrospectively and included a relatively small number of isolates. The small sample size in our multivariable analysis resulted in wide confidence intervals for certain predictors; therefore, these identified risk factors for CHR should be interpreted with caution and validated in larger cohorts. Second, our analysis was based on culture-dependent methods and did not incorporate molecular or metagenomic approaches that could better characterize microbial population diversity. Therefore, the underlying mechanisms driving heteroresistance and its relationship with the broader bloodstream microbial environment remain to be elucidated. Third, PAP experiments were performed once for each isolate and formal reproducibility or inter-run variability analyses were not performed. Finally, the classification of stable and unstable heteroresistance was based on a seven-day serial passage protocol and should be interpreted in the absence of a standardized definition for heteroresistance stability.

## Conclusions

In conclusion, we identified a remarkably high prevalence of colistin heteroresistance among colistin-susceptible *P. aeruginosa* bloodstream isolates. GT failed to detect any heteroresistant isolate, highlighting the limitations of conventional susceptibility testing methods for identifying resistant subpopulations. Although CHR was not associated with mortality in our cohort, its high prevalence and potential implications for antimicrobial therapy warrant further attention. Future studies incorporating larger patient cohorts and molecular investigations are needed to better define the clinical significance, epidemiology, and underlying mechanisms of CHR in *P. aeruginosa* bloodstream infections.

## Data Availability

Data available from the corresponding author on reasonable request.
